# Patterns of Eating Behavior among 13-Year-Old Adolescents and Associated Factors: Findings from the Generation XXI Birth Cohort

**DOI:** 10.3390/healthcare11101371

**Published:** 2023-05-10

**Authors:** Ingrid Nakamura, Andreia Oliveira, Sarah Warkentin, Bruno M. P. M. Oliveira, Rui Poínhos

**Affiliations:** 1 Faculdade de Ciências da Nutrição e Alimentação, Universidade do Porto, Rua do Campo Alegre, 823, 4150-180 Porto, Portugal; nakamura.ingrid@gmail.com (I.N.); bmpmo@fcna.up.pt (B.M.P.M.O.); 2 EPIUnit—Instituto de Saúde Pública, Universidade do Porto, Rua das Taipas, 135, 4050-600 Porto, Portugal; acmatos@ispup.up.pt (A.O.); sarah.warkentin@isglobal.org (S.W.); 3 Laboratório para a Investigação Integrativa e Translacional em Saúde Populacional (ITR), Universidade do Porto, Rua das Taipas, 135, 4050-600 Porto, Portugal; 4 Faculdade de Medicina, Universidade do Porto, 4200-319 Porto, Portugal; 5 ISGlobal, 08036 Barcelona, Spain; 6 Laboratório de Inteligência Artificial e Apoio à Decisão, Instituto de Engenharia de Sistemas e Computadores—Tecnologia e Ciência, Campus da Faculdade de Engenharia, Universidade do Porto, 4200-465 Porto, Portugal

**Keywords:** eating behavior, adolescents, depression, latent class analysis, environmental influences, weight

## Abstract

Eating behavior adopted during adolescence may persist into adulthood. The aims of this study were to identify eating behavior patterns among Portuguese adolescents and to explore whether groups differ in terms of early life and family characteristics, severity of depressive symptoms, and body mass index (BMI) z-score. Participants were 3601 13-year-olds enrolled in the birth cohort Generation XXI. Eating behavior was assessed using the self-reported Adult Eating Behavior Questionnaire (AEBQ), validated in this sample. The severity of depressive symptoms was measured through the Beck Depression Inventory (BDI-II), and data on sociodemographic and anthropometrics were collected at birth and 13-years-old. Latent class analysis was conducted, and associations were estimated using multinomial logistic regression models. Five patterns of individuals were identified: “Picky eating”, “Disinterest towards food”, “Food neophilia”, “Emotional eating”, and “Food attractiveness”. The adolescents’ sex, maternal education, BMI z-score, and severity of depressive symptoms were significantly associated with the identified patterns. In particular, adolescents with a higher BMI z-score were more likely in “Food neophilia” while individuals with more severe depressive symptoms were in the “Picky eating”, “Emotional eating”, and “Food attractiveness” patterns. These findings suggest a starting point for the development and planning of targeted public health interventions.

## 1. Introduction

Adolescence is a transitional period from childhood into adulthood marked by considerable physiological, psychological, and social changes. Lifestyles learned and adopted during adolescence may persist into adulthood, such as eating behavior [1]. Eating behavior reflects the actions related to eating and include, for example, where, when, and with whom we eat [2] and involve traits related to appetite, food acceptance, and meal patterns [3], with one’s appetite being influenced by a complex gene–environment interaction [4].

Some eating behaviors are related with the greater interest towards foods and drinks, the so-called food approach behaviors, which are generally positively associated with body mass index (BMI) z-scores among children [5,6,7]. Other behaviors, the so-called food avoidant behaviors, are related with the avoidance or lack of interest towards foods and drinks and are commonly negatively associated with child BMI z-scores [5,6,7]. It is important to mention that opposite pathways may also be true, with the BMI z-score in childhood prospectively predicting, years later, greater scores on the food approach and lower scores on the food avoidant eating behaviors [8,9]. The BMI z-score may also predict greater disordered eating, such as binge eating, during adolescence [10].

Although these eating behaviors have been shown to be moderately stable across childhood [11,12], Hunot-Alexander et al. (2019) [13] found that older adolescents (aged 14 to 18 years), compared to younger (aged 11 to 13 years), were more susceptible to external food cues and enjoyed food more, thus showing heightened food approach behaviors, which may enhance the risk of weight gain [13,14]. Although these behaviors may compromise adolescent health, the exploration of the patterns and trajectories of adolescent eating behavior in the literature remains limited.

Besides BMI, other relevant factors may also influence adolescent eating behaviors, such as mental health problems, given the fact that adolescence confers significant vulnerability to psychological problems [15]. Depression is a mental illness recognized as a leading cause of illness and disability among this population [16], and adolescents who are depressed may change their appetite and dietary patterns, resulting in loss weight or weight gain and obesity [17,18].

Additionally, the familial environment could also affect, in a great extension, adolescent eating behaviors. Children and adolescents whose mothers had a lower age, educational level, and weight tended to have a greater risk of developing picky eating behaviors (rejection of a variety of both familiar and unfamiliar foods) [19,20]. In other studies, those who had siblings showed a lower risk of being characterized as picky eaters [21], and those with lower income were more likely to overeat (of an objectively large amount of food) [22,23].

In childhood, researchers have associated characteristics in the beginning of life, such as a low birth weight [24], preterm birth, and lower fetal growth [25], with later eating difficulties and a picky eating behavior pattern. In adolescents, little is known on how these early life characteristics may relate with later eating behavior patterns. It is plausible to assume that similar patterns may occur in adolescence based on the evidence that eating behaviors are stable throughout childhood [11,12] and may persist through adolescence [13,26]; however, research is necessary to confirm this hypothesis.

Considering the great number of environmental influences on adolescent eating behaviors, and, given the fact that studies in this field are still scarce, a deeper understanding of eating behavior patterns and its determinants among adolescents is warranted. The identification of eating behavior patterns could help in the identification of risk groups, and in the development and planning of public health interventions aiming to improve eating behavior throughout life and, consequently, to prevent excess weight gain.

In this sense, the aims of this study are (i) to identify groups of individuals with similar patterns of eating behavior in a large sample of 13-year-old adolescents of an ongoing population-based cohort study and (ii) to explore whether the identified groups differ in terms of early life characteristics, family environment, severity of depressive symptoms, and BMI z-score.

## 2. Materials and Methods

### 2.1. Study Design and Population

This study included participants enrolled in Generation XXI, an ongoing population-based birth cohort from Porto, northern Portugal [27]. Recruitment took place between April 2005 and August 2006, and mothers were invited to participate in the study within 72 h after delivery in all public maternities in the metropolitan area through face-to-face interviews. Of all eligible mothers, 91% agreed to participate (8495 mothers and 8647 children at baseline). All families were invited for follow-ups at 4 (*n* = 7459, 86%), 7 (*n* = 6889, 80%), 10 (*n* = 6397, 76%), and 13 years of age (*n* = 4643, 55%—lower than expected due to an earlier stop due to the COVID-19 pandemic). Additional details about the recruitment and sample can be found elsewhere [27,28].

**Figure 1:**
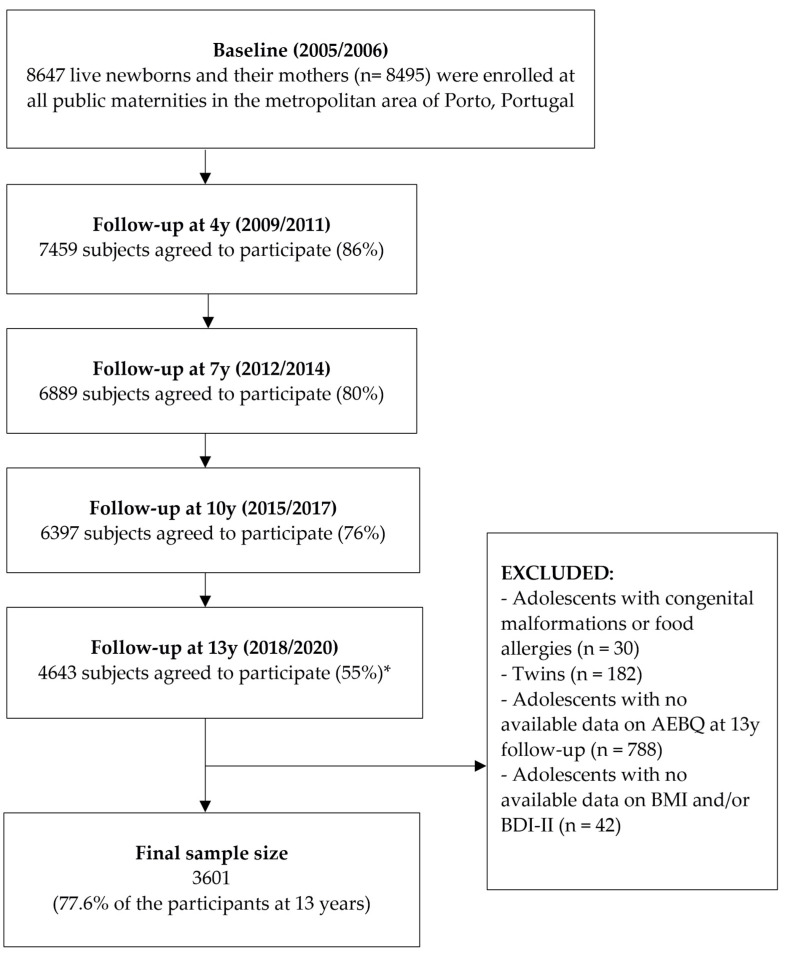
Study flowchart from the Generation XXI birth cohort. * Follow-up with an earlier stop due to the COVID-19 pandemic.

The current study included 3601 adolescents (77.6% of the participants at 13 years) with available data on self-reported eating behaviors and severity of depressive symptoms at the 13-year follow-up and with other data of interest (see study flowchart in Figure 1).

Generation XXI was approved by the University of Porto Medical School/S. João Hospital Centre Ethics Committee (27 April 2005) and by the Portuguese Data Protection Authority (Protocol code 5833, approved on 30 May 2011). All the phases of the study complied with the Ethical Principles for Medical Research Involving Human Subjects expressed in the Declaration of Helsinki. Written informed consent from the parents (or legal substitute) and oral assent from the children/adolescents were obtained in each evaluation.

### 2.2. Measures

#### 2.2.1. Adolescent Self-Reported Eating Behavior

Adolescent eating behavior was assessed at 13 years of age using a self-reported questionnaire, the Adult Eating Behavior Questionnaire (AEBQ). The AEBQ was originally developed by Hunot-Alexander et al. (2019) [13] among adolescents in the UK and was based on the widely used Children’s Eating Behavior Questionnaire (CEBQ) [29]. It contains 35 items, originally divided into eight subscales, conceptually grouped into food approach and food avoidance behaviors [13,29,30].

The four AEBQ food approach subscales are Hunger (5 items, e.g., “I often feel hungry”), Food responsiveness (4 items, e.g., “I am always thinking about food”), Emotional overeating (5 items, e.g., “I eat more when I’m anxious”), and Enjoyment of food (3 items, e.g., “I love food”). The remaining subscales, described as food avoidant dimensions, include Satiety responsiveness (4 items, e.g., “I often leave food on my plate at the end of a meal”), Emotional undereating (5 items, e.g., “I eat less when I’m worried”), Food fussiness (5 items, e.g., “I refuse new food at first”), and Slowness in eating (4 items, e.g., “I eat slowly”). All items were responded using a 5-point Likert scale, ranging from 1 = “strongly disagree” to 5 = “strongly agree”. Nonetheless, in accordance with the original scale, five of the items had reversed scoring [13].

Internal consistency was adequate for all AEBQ subscales (Cronbach’s α ranging from 0.66 to 0.88) [13]. In Portugal, Warkentin et al. (2022) [30] validated the AEBQ among 13-year-old adolescents from the Generation XXI cohort. The adapted version of the AEBQ contains 30 items and showed a different factor structure (5 subscales, namely Food responsiveness/Enjoyment of food, Emotional overeating, Slowness in eating, Food fussiness, and Emotional undereating) and an adequate internal consistency (Cronbach’s α ranging from 0.77 to 0.89).

#### 2.2.2. Severity of Depressive Symptoms

The severity of depressive symptoms at 13-years-old was measured using a validated updated version of the former Beck Depression Inventory (BDI) [31], which was originally proposed by Beck et al. (1961), namely the BDI-II [32].

The BDI-II is a 21-item self-report inventory, in which all items are rated on a 4-point Likert scale ranging from 0 to 3 and related to progressive gravity of specific symptoms (e.g., “I do not feel sad” (0), “I feel sad much of the time” (1), “I am sad all the time” (2), and “I am so sad or unhappy that I can’t stand it” (3)). Adolescents were asked to select the best answer capable of describing how they had felt in the previous two weeks, including the day of the evaluation.

The total score, resulting from the sum of all items, ranges from 0 to 63. A score of lesser than or equal to 13 represents minimal depressive symptomatology, 14 to 19 represents mild depressive symptoms, 20 to 28 represents moderate depressive symptoms, and scores equal to or greater than 29 indicate severe depressive symptoms [32].

In Portugal, Coelho et al. (2002) [33] and Brochado et al. (2008) [34] investigated the BDI-II psychometric properties, and this tool appeared to be an adequate measure of depressive symptoms in adolescents, with a good internal consistency (Cronbach’s α = 0.89 in both studies).

#### 2.2.3. Sociodemographic and Anthropometric Characteristics

Data on sociodemographic characteristics, such as maternal age, education, household monthly income, number of children, child sex, birth weight, and gestational age (according to ultrasound) were collected at baseline through face-to-face interviews conducted by trained researchers using structured questionnaires.

At the 13-year-old follow-up, data on the number of siblings who lived with the adolescent and respective ages were collected. These data were combined with the data on the number of children (baseline) and used to classify the adolescents as “only child”, “oldest sibling”, “middle sibling”, or “youngest sibling” (classification of “birth order”).

Weight for gestational age was calculated and classified according to the sex-specific population-based Kramer growth standards [35]. Small for gestational age was defined as below the 10th percentile, large for gestational age above the 90th percentile, and values within these thresholds were deemed to be adequate for gestational age. These references refer only to single births.

Maternal height and weight before pregnancy were self-recorded at birth, and BMI was calculated and classified according to the World Health Organization (WHO) cut-offs [36].

Adolescent anthropometrics, i.e., weight and height, were measured by trained staff, according to standard procedures. Adolescents were weighted in underwear and without shoes by trained researchers using a digital scale, and the measure was recorded to the nearest 0.1 kg. Height was also measured without shoes using a fixed stadiometer to the nearest 0.1 cm. These measurements were used to calculate age- and sex-specific BMI z-scores, according to WHO [37]. Underweight was classified for those adolescents with BMI standard z-scores < −2, normal weight for BMI z-scores in [−2; +1], overweight for BMI z-scores in [+1; +2], and obesity for BMI z-scores > +2.

### 2.3. Statistical Analyses

Descriptive statistics were computed using means and standard deviations or medians and percentiles (P25; P75) for continuous variables based on the distribution of the variables and using frequency distribution for categorical variables.

In this study, latent class analysis (LCA) was conducted using the procedure poLCA 1.4.1 in R 4.1.3 and RStudio 22.02.1. LCA belongs to the person-centered approaches [38] (in opposition to factor analysis that presents groups of variables) [39], and it was used to identify groups of individuals with similar patterns of eating behaviors among the 13-year-olds based on responses given by the adolescents to the AEBQ. LCA used the 30 items from the AEBQ adapted version, and in this analysis, we did not consider the previously reported factorial structure of the data. The central idea of the LCA is to recover hidden groups from observed data, which are useful to categorize a large number of individuals into a few subgroups in which the observations of several categorical variables are similar to each other but different from those in other subgroups [38,40]. Although LCA and Factorial Analysis share some similarities, LCA focus on the structure of groups determined by a latent (unobserved) multinomial variable while Factorial Analysis is based on the correlations between the observed variables [39].

In order to select the most robust and adequate number of latent classes, different values of classes (1 to 14) were evaluated, and the procedure was repeated 100 times for each class [38].

The most parsimonious number of latent classes was determined by examining the model fit, namely the Bayesian information criterion (BIC) [40]. Furthermore, an additional fit criteria was considered while choosing the class, namely the elbow heuristic for the BIC plot, which means finding the “elbow” of the plot, after which the decline in the value of these indicators reaches a plateau and becomes less noticeable, suggesting the optimal solution on elbow plot [41].

Resulting classes were compared in terms of the responses given by the adolescents to each item from the Portuguese version of the AEBQ using Kruskal–Wallis test, and the mean ranks (mR) were converted to mean percentiles (mP) using the formula mP = 100% × (mR—1)/(*n*—1).

Univariate and multivariable multinomial logistic regression models were run using IBM SPSS Statistics version 28.0 for Windows to compute crude and adjusted odds ratios (OR) and 95% confidence intervals (95% CI) estimating the association between the latent classes and adolescent sex, severity of depressive symptomatology, BMI z-score, weight for gestational age, birth order, maternal age, education and BMI before pregnancy, and household monthly income. Statistical significance was set at 5%, and all tests were two-tailed.

## 3. Results

The total sample consisted of 3601 adolescents (50.8% were boys) with a median age of 13.3 years (P25 = 13.2; P75 = 13.4). The mean BMI z-score of participants was 0.44 (SD = 1.16), and nearly a third (33.1%) had excess weight (overweight/obesity). At baseline, family income was ≤1000 euros per month in 31.8% (*n* = 1010) of households, and mothers were, on average, 30.1 years old (SD = 5.0) and had 11.3 years (SD = 4.3) of schooling. Regarding the severity of the depressive symptomatology, 15% (*n* = 540) of adolescents showed mild to severe depressive symptoms. Further and more detailed sample characteristics are displayed in Table 1.

### 3.1. Latent Class Analysis for the Identification of Adolescent Eating Behavior Patterns

**Figure 2:**
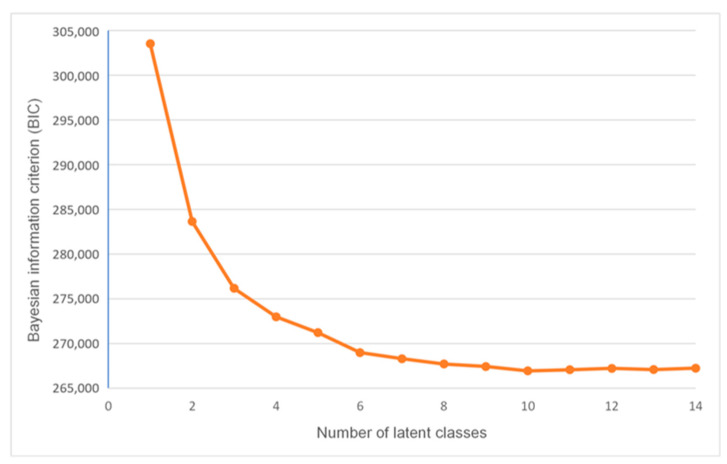
Elbow plot for 1- to 14-class solutions for eating behaviors among 13-year-olds.

The model fit for 1 to 14 class solutions was conducted. The lowest value of BIC supported the 10-class solution. However, according to the elbow plot, as shown in Figure 2, the 5-class solution showed to be the most adequate and parsimonious model, compared to the 10-class solution (albeit the gradually decreasing values of BIC up to 10 classes). Therefore, the 5-class (or 5 patterns) solution was selected to best represent the current data.

**Figure 3:**
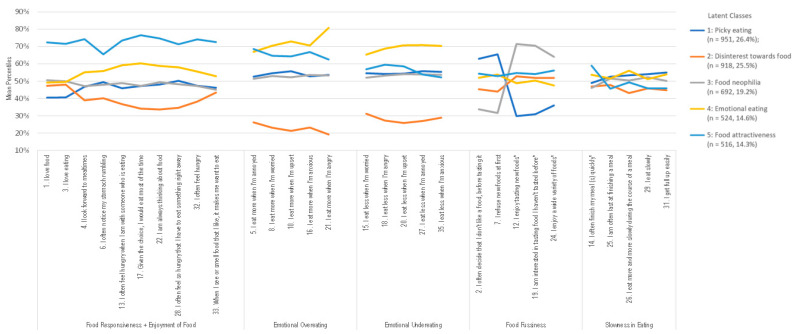
Visual description of the characteristics of the five adolescent eating behavior patterns derived from the latent class analysis of the Portuguese AEBQ items.

Figure 3 graphically displays the characteristics of the five patterns of eating behaviors among the 13-year-olds. The first pattern, accounting for 26.4% of the adolescents in this sample, was mainly characterized by those who agreed with the items of Food fussiness (#2 “I often decide that I don’t like a food before tasting it” and #7 “I refuse new foods at first”) and, at the same time, by those who disagreed with the items #12 (“I enjoy tasting new foods”), #19 (“I am interested in tasting food I haven’t tasted before”), and #24 (“I enjoy a wide variety of foods”) from the same subscale. They also disagreed with the items #1 (“I love food”) and #3 (“I love eating”) from Food responsiveness/Enjoyment of food subscale of the Portuguese version of the AEBQ and agreed with the items #29 (“I eat slowly”) and #31 (“I get full up easily”) from Slowness in eating subscale. This pattern was labeled “Picky eating” since the adolescents in this group were less prone to show an interest in trying novel and unfamiliar foods; in addition, they showed a pattern of slow and joyless eating.

The second pattern, accounting for 25.5% of participants, was predominately described by the disagreement with all items that compose the Emotional overeating and Emotional undereating subscales and with seven out of ten items from Food responsiveness/Enjoyment of food subscale (#4 “I look forward to mealtimes”, #6 “I often notice my stomach rumbling”, #13 “I often feel hungry when I am with someone who is eating”, #17 “Given the choice, I would eat most of the time”, #22 “I am always thinking about food”, #28 “I often feel so hungry that I have to eat something right away”, and # 32 “I often feel hungry”). Furthermore, they were also more prone to disagree with the items #26 (“I eat more and more slowly during the course of a meal”), #29 (“I eat slowly”), and #31 (“I get full up easily”) from the Slowness in eating subscale. Due to the disagreement with almost all subscales except for the Food fussiness that may indicate a reduced general interest in food, this group was labeled “Disinterest towards food”.

Individuals in the third pattern, which accounted for 19.2% of this sample, in contrast to those adolescents in the “Picky eating” pattern, were more prone to agree with the items #12 (“I enjoy tasting new foods”), #19 (“I am interested in tasting food I haven’t tasted before”), and #24 (“I enjoy a wide variety of foods”) from the Food fussiness subscale. They were also prone to disagree with two items of the same subscale (# 2 “I often decide that I don’t like a food, before tasting it” and #7 “I refuse new foods at first”), indicating that adolescents in this pattern were more likely to be willing to try novel foods; thus, this group was named “Food neophilia”.

About 14.6% of adolescents were assigned to the fourth pattern. These adolescents were more likely to agree with the items of Slowness in eating subscale (#26 “I eat more and more slowly during the course of a meal” and #31 “I get full up easily”). Nonetheless, this class was predominately described by the concordance with all items that compose the Emotional overeating and Emotional undereating subscales. Consequently, this group was labeled “Emotional eating”.

The fifth pattern included 14.3% of the adolescents who were more likely to agree with the item #10 (“I eat more when I’m upset”) from Emotional overeating subscale. They were also likely to agree with the item #14 (“I often finish my meal(s) quickly”) from Slowness in eating subscale and to disagree with the items #29 (“I eat slowly”), #31 (“I get full up easily”), and #25 (“I am often last at finishing a meal”) from the same subscale. However, this group was predominately described by the agreement with all items that compose the Food responsiveness/Enjoyment of food subscale, indicating that individuals in this pattern are more likely to be willing to suggest a movement toward or desire for food; thus, this class was labeled “Food attractiveness”. In Table 2, we present the mean percentile per AEBQ subscale for each latent class. The values for the food fussiness subscale were computed after reversing items 12, 19, and 24.

### 3.2. Associations between Adolescent Eating Behavior Patterns and Sociodemographic and Anthropometric Characteristics

In Table 3, univariate multinomial logistic regression models between adolescent eating behavior patterns, derived from the LCA, and various sociodemographic and anthropometric characteristics are described. “Disinterest towards food” (Class 2) was chosen as the reference pattern for easiness of comparisons, as it refers to the overall higher disagreement with the sentences. Adolescents whose mothers had higher age at baseline and higher household income had lower chance of belonging to the “Food attractiveness” group, compared to the reference pattern (*p* < 0.001 for both). However, in the multivariable multinomial logistic regression models, after further adjusting for maternal and adolescent characteristics, these associations were no longer significant (displayed in Table 4 and Table 5, respectively).

Maternal education, adolescent sex, BMI z-score, and severity of depressive symptoms showed to be significantly associated with the identified patterns of eating behaviors (Table 3; *p* < 0.001 for all), and these associations were maintained after further adjustments (Table 4 and Table 5; *p* < 0.001, except for maternal education after adjustment for adolescent characteristics, for which *p* = 0.005).

In the final adjusted model, compared to boys, girls were significantly more likely to belong to the “Picky eating”, “Food neophilia”, “Emotional eating”, and “Food attractiveness” patterns compared to the reference category “Disinterest towards food” (OR 1.252, 1.527, 1.611, and 1.672, respectively). Each 1-unit increase in BMI z-score was associated with an increase of 18% to be in the “Food neophilia” class, and those whose mothers had a higher educational level were less likely to be in the latent class of “Food attractiveness”, compared to the “Disinterest towards food” class. Adolescents with more severe depressive symptoms (i.e., higher scores on BDI-II) were more likely to belong to the “Picky eating” (OR: 1.022), “Emotional eating” (OR: 1.052), and “Food attractiveness” (OR: 1.098) patterns compared to the reference class.

No significant associations (*p* > 0.05) were found between eating behavior patterns and weight for gestational age, BMI before pregnancy, or birth order.

## 4. Discussion

To our knowledge, this study is the first to characterize and identify groups of individuals with similar patterns of eating behavior in a large sample of 13-year-old Portuguese adolescents of an ongoing population-based birth cohort study using latent class analysis, a person-centered approach. Five patterns were identified, namely “Picky eating”, “Disinterest towards food”, “Food neophilia”, “Emotional eating”, and “Food attractiveness”, with distinct characteristics between them. We found that adolescent sex, maternal education, BMI z-score, and severity of depressive symptoms were associated with the identified latent classes.

Among the five eating behavior patterns, about 26.4% of adolescents were categorized in the “Picky eating” class, which has a similar prevalence to the one observed in another Portuguese study [22] that found a picky eating frequency of 23.1% in a school-aged sample with, on average, 10.8 years of age. Our finding may suggest that picky eating behavior was relatively frequent in this age group, although picky eating has been reported, in most cases, as a transient behavior, having the highest prevalence around 2 years of age, followed by a decline until 6 years of age [42,43]. However, there is also evidence that this eating behavior can be found across the whole lifespan, from childhood through adulthood [26,43,44], which is in line with our finding.

While it is well established that some degree of picky eating is considered a part of normal child development and not necessarily warranting clinical attention, little is known on how this behavior may impact diet, growth, and health outcomes [45]. In 2020, Herle et al. [26], found associations between picky eating behaviors in childhood and an increased risk of developing anorexia nervosa later in life. The mechanisms behind this association are still unknown, and future research is necessary.

It is important to mention that individuals of the current study grouped in the “Picky eating” pattern, besides showing a lower interest in trying novel and unfamiliar foods, also had a lower enjoyment when eating a variety of foods and eat slower and got full more easily. This finding was similar with the study by De Barse et al. five years earlier [46], which used the CEBQ and identified a pattern with children who scored high on the CEBQ’s food fussiness and who also got full more easily and showed a pattern of slow and joyless eating. Nonetheless, the authors suggested that the combination of those behaviors could reflect a more “severe” fussy eating pattern (another form to refer to ‘picky’ eating) once they found that this pattern may result in a possible adverse effect on healthy growth associated with the risk of developing underweight and relatively low fat-free mass in children [46].

Thus, further attention to the “Picky eating” pattern found in our study is warranted. Additional research may help to identify whether subpopulations exist within picky eating profiles that are clinically important to differentiate.

We found that adolescents with a higher BMI z-score also belonged to the “Food neophilia” pattern. This association was unexpected since children and adolescents in heavier weight groups usually tend to show greater emotional eating, food responsiveness, and enjoyment of food behaviors [47,48]. However, considering that neophiliac individuals tend to like and approach new foods [49], indicating an eating behavior that involves a movement towards or desire for food, it is plausible to assume that the food neophilia trait may relate with food approach eating behaviors as well. Previous studies have suggested that food approach behaviors are positively related to one another [13,50] and are generally positively associated with BMI [5,6,7,47], which may justify our result.

Furthermore, research on food neophilia and its possible health effects remains limited [51]. On the other side, there is a vast amount of information on food neophobia (the tendency to avoid or refuse novel or unfamiliar foods), considered to be a specific component of picky eating behavior [19,45,51,52].

However, the majority of studies found no significant associations between food neophobia and weight status, and studies specifically focused on adolescence, the literature is also quite sparse [45,51,52]. In a systematic review [52] examining the association between food neophobia and weight status, just one out of seven studies found a positive association with overweight, contradicting our finding [53]. Nevertheless, the age group was not the same as our sample (children aged 2 to 6 years).

It is important to mention that individuals in the “Food neophilia” pattern, besides being more prone to show an interest in trying novel and unfamiliar foods, were also more prone to enjoy a wide variety of foods. Nonetheless, dietary variety has been recognized as a paradox [54]: although the consumption of a variety of food items has been recommended to promote an adequate nutrient intake [55], studies have also associated dietary variety with a higher energy intake and weight gain [56,57]. The idea of variety and diversity may lead to the inclusion of unhealthy foods in the diet, increasing, consequently, the intake of energy, processed foods, sugar-sweetened beverages, and refined grains [56,57].

In the current study, the association between the “Food neophilia” pattern and energy intake was not investigated; however, there might be another explanation for the association found between the “Food neophilia” class and adolescents with a higher BMI z-score. This may be considered a starting point for future research aiming to examine associations between the AEBQ subscales and measures of food preferences, intake, and diet quality in adolescents. Moreover, further studies are also warranted aiming to clarify the association between individuals with the “Food neophilia” eating pattern and potential health outcomes.

We found that some patterns, such as “Picky eating”, “Food neophilia”, and, especially, “Emotional eating” and “Food attractiveness”, included mainly girls. Contrarily to our findings, Loh et al. (2013) [58], using the CEBQ, found no significant differences in eating behaviors between Malaysian adolescent boys and girls while Santos et al. (2011) [6] observed in a sample of Chilean 6- to 12-year-olds significantly higher scores of the food avoidant subscales slowness in eating, satiety responsiveness and emotional under-eating in girls compared to boys.

Nonetheless, Hunot-Alexander et al. 2019 [13] reported that girls showed higher scores on emotional overeating, satiety responsiveness, and slowness in eating. Consistent with these findings, a Polish study observed in a sample of secondary school students that girls, besides reporting higher levels of emotional overeating, satiety responsiveness, and slowness in eating, also showed higher scores of food responsiveness and emotional undereating [50]. In both studies, boys did not show higher scores for any of the subscales, which is in line with our findings, wherein boys showed to be more likely to disagree with almost all subscales [13,50].

In a Chinese study [40] with young adults using LCA, it was observed that women, compared to men, were more likely to be in the patterns of “Emotional overeating” and “Emotional over- and undereating”. The “Emotional over- and undereating” was a mixed pattern of individuals identified with both emotional over–undereating behaviors, which was considered the most problematic pattern, with the highest level of eating disorder symptoms and psychological distress. The possible explanation for this finding was that individuals in this pattern may be more likely to have difficulties in emotional regulation, which also can result in more eating disorder symptoms [40].

Interestingly, we also found a mixed pattern in our study, namely “Emotional eating”, wherein, as previously mentioned, adolescents were more prone to both overeat *and* to undereat in response to emotions. In line with our finding, in the previous validation study of the CEBQ-P with children aged 7 years old from the Generation XXI cohort [59], these two emotional eating subdomains loaded into the same factor, obtained from a Principal Component Analysis, showing a high correlation between them. Likewise, a cohort study of 4-year-old British twins [60] also observed a positive correlation, indicating that some children tend to both overeat and undereat as a response to emotions and stress.

The identification of such a mixed pattern suggests that emotional over- and undereating could be two unique constructs that may co-occur among individuals [40]. However, the explanation regarding emotion regulation should be explored in future studies, as well as a better understanding of the “Emotional eating” pattern and its characteristics. Furthermore, a more comprehensive understanding of sex differences for certain eating behaviors in adolescence are needed using the subscales of the more recent and validated AEBQ [30].

In the current study, the prevalence of adolescents with mild to severe depressive symptoms was 15%, higher than the frequency observed in another Portuguese study [61], which reported 11.9% among adolescents with the same age. In addition, we found that adolescents with higher scores on BDI-II had higher odds of belonging to the “Picky eating” and, especially, “Emotional eating” and “Food attractiveness” patterns. This association may be, at least in part, explained by the fact that individuals with depressive symptoms have a dysfunctional coping strategy that can lead to the development of abnormal eating behaviors, such as emotional eating, wherein food is used as a natural reward or gratification habit for alleviating and dealing with stress and negative feelings [62,63].

Different studies have suggested an association between emotional eating and higher depressive symptoms [63,64]. In an Australian study [65], for example, the authors found that adolescents (aged 10 to 13 years) who reported more emotional eating concurrently reported higher depressive symptoms. For that reason, we expected that the “Emotional eating” pattern would include adolescents with the highest scores on BDI-II. In addition, considering that the “Emotional eating” was a mixed pattern, as stated above, supposedly, the individuals in this class could have difficulties regulating their emotions.

Nonetheless, it has also been reported that some individuals, in order to shift attention away from negative feelings, such as depressive feelings, may focus their attention at the current and immediate environment stimulus, as accessible food cues, for example [66]. Considering that the “Food attractiveness” pattern included adolescents who were more prone to respond to external food cues, it may justify the association found between this pattern and adolescents with more severe depressive symptoms in our study. However, further studies are necessary to examine this possible link using measures that specifically address adolescent’s responsiveness to external cues, such as visual, environmental, or social cues.

Lastly, we found that adolescents whose mothers had a higher educational level were less likely to be in the latent class of “Food attractiveness”. Contrary to this finding, in the original AEBQ validation study [13], no association between maternal education and adolescent eating behavior was observed. In the previous validation study of the CEBQ-P with children aged 7-years-old from the Generation XXI cohort [59], the authors found that a higher maternal education (≥12 vs. <9 years) was associated with higher appetite disinhibition scores (more related to subdomains measuring external food cues and emotional responses toward foods, which included food responsiveness).

However, in agreement with the current result, a previous study explored the differences between children’s eating behaviors (aged 6 to 10 years) and their relationship with maternal education level, and it was observed that individuals whose mothers had a bachelor’s degree or graduate level education, compared to those with a lower education level, showed significantly decreased CEBQ-Food responsiveness [67].

In addition, a study with Chinese students aged 11 to 13 years [68], using the Dutch Eating Behavior Questionnaire (DEBQ) [69], showed that higher parental education and family income were associated with higher levels of restrained eating (the tendency to restrict food intake aiming to control the body weight). Considering that the restrained eating subscale is related with undereating, it is likely to be inversely related to the AEBQ food approach dimensions, confirming our result.

It is important to note that, besides this association between the “Food attractiveness” pattern and maternal educational level, this pattern, as we had mentioned before, was also associated with a higher score of depressive symptoms. Interestingly, a previous study conducted with children and adolescents showed that participants whose mothers had higher levels of education reported less mental health problems compared with those of mothers with lower education [70]. This finding is consistent with that reported in a study carried out in Canada on participants aged 12 to 24 years, wherein maternal low education (mothers with less than secondary school education) was associated with an increased risk of depressive episodes among their offspring [71].

Furthermore, starting at puberty, girls are at the greatest risk for depression, with higher prevalence compared to boys [72]. In our study, as described above, girls showed more frequently the “Food attractiveness” pattern compared to boys. Thus, these associations that we found with potential risk factors emphasize that further attention to this pattern is warranted. Indeed, more research is also required to obtain a thorough understanding of the effects of maternal educational levels on adolescent eating behavior.

The strengths of this study lie in the use of AEBQ recently validated among Portuguese adolescents, providing a convenient and easy-to-use tool in this age group in large-scale research [30]. The BDI-II is also considered a reliable and well-validated adolescent report as well [73]. Strengths also include the large sample size, the execution of standardized assessments, and the inclusion of objectively measured weight and height in adolescents. It is important to highlight that, by using a person-centered approach (LCA), our study was able to identify the existence of an unobserved subgroup with individuals with both emotional overeating and emotional undereating (likewise the finding observed in the CEBQ-P validation study from the Generation XXI cohort, as mentioned before), which should be explored in further investigations.

The current study has some limitations that need to be addressed. First, adolescent eating behavior was assessed using a self-reported questionnaire, which may be subject to social desirability bias. Additionally, other aspects that may have influenced adolescent eating behavior were not included, such as food preferences, intake, diet quality, and psychological problems other than depression, which are beyond the scope of this study. Second, the severity of depressive symptoms was also obtained through a self-reported questionnaire, which may also be affected by social desirability bias. Furthermore, it is important to highlight that this measure does not permit a diagnostic assessment of depression as would a clinical interview. Finally, as mentioned before, the follow-up was interrupted due to the COVID-19 pandemic.

## 5. Conclusions

By using LCA, the current study identified five distinct groups of individuals with similar patterns of eating behavior in a large sample of 13-year-old adolescents of an ongoing population-based cohort study: “Picky eating”, “Disinterest towards food”, “Food neophilia”, “Emotional eating”, and “Food attractiveness”. Some patterns, such as “Picky eating”, “Food neophilia”, and, especially, “Emotional eating” and “Food attractiveness”, included mainly girls. Adolescents whose mothers had a higher educational level were less likely to be in the latent class of “Food attractiveness”, and those with a higher BMI z-score were more likely to belong to the “Food neophilia” pattern. Individuals with more severe depressive symptoms had higher odds of belonging to the “Picky eating” and, especially, “Emotional eating” and “Food attractiveness” patterns.

Considering the great amount of environmental influences on adolescent eating behaviors, our findings provide a starting point for the development and planning of targeted public health interventions. Given the novelty of these findings, future research is needed to clarify whether these eating behavior patterns are associated with health outcomes, and it would be interesting to explore how these eating behaviors evolve across age, from childhood to adolescence, using a longitudinal perspective.

## Figures and Tables

**Table 1:** Mother’s and adolescent’s characteristics at baseline and follow-up at 13-years-old (*n* = 3601).

	Mean (SD)/ Median (P25; P75)	*n* (%)
***Maternal characteristics***		
**Age at baseline** (years)	30.1 (5.0)	
**Education at baseline** (years)	11.3 (4.3)	
**Household income** (€/month) [*n* = 3180]		
≤1000		1010 (31.8)
1001 to 2000		1591 (50.0)
>2000		579 (18.2)
**BMI before pregnancy** (kg/m^2^) *	23.0 (21.0; 25.8)	
**Weight status (BMI) before pregnancy**		
Underweight		107 (3.2)
Normal weight		2201 (65.5)
Overweight		750 (22.3)
Obesity		303 (9.0)
***Birth characteristics***		
**Birth weight** (g)	3225 (2950; 3510)	
**Gestational age** (weeks) [*n* = 3599]	39 (38; 40)	
**Weight for gestational age** ** [*n* = 3599]		
Small		482 (13.4)
Adequate		2979 (82.7)
Large		138 (3.8)
***Adolescent characteristics***		
**Sex**		
Male		1831 (50.8)
Female		1770 (49.2)
**Age** (years)	13.28 (13.22; 13.39)	
**BMI z-score** ***	0.44 (1.16)	
**Weight status (BMIz)**		
Underweight		74 (2.1)
Normal weight		2334 (64.8)
Overweight		851 (23.6)
Obesity		342 (9.5)
**Birth order**		
Only child		1137 (31.6)
Oldest sibling		946 (26.3)
Middle sibling		192 (5.3)
Youngest sibling		1326 (36.8)
**Portuguese version of the Adult Eating Behavior** **Questionnaire (AEBQ)** (range: 1 to 5) [30]		
Food Responsiveness/Enjoyment of Food	2.95 (0.64)	
Emotional Overeating	2.14 (0.79)	
Emotional Undereating	2.40 (0.94)	
Food Fussiness	2.77 (0.86)	
Slowness in Eating	2.62 (0.78)	
**Beck Depression Inventory II (BDI-II)** [32]	5 (2; 10)	
**Severity of depressive symptoms**		
Minimal depressive symptomatology (range: 0 to 13)		3061 (85.0)
Mild depressive symptoms (range 14 to 19)		318 (8.8)
Moderate depressive symptoms (range 20 to 28)		146 (4.1)
Severe depressive symptoms (range 29 to 63)		76 (2.1)

M: Mean, SD: Standard deviations, Md: Median, P: Percentiles, BMI: Body mass index, BMIz: Body mass index z-score. * Maternal weight status categories were defined according to the WHO Standards for BMI (kg/m^2^): Underweight: BMI < 18.5, Normal weight: BMI in [18.5; 25.0[ Overweight: BMI in [25.0; 30.0[, Obesity: ≥ 30.0 [36]. ** Weight for gestational age was classified according to the Kramer Growth Standards, as follows: Small: <P10, Adequate: between P10 and P90, Large: >P90 [35]. *** Adolescent weight status categories were defined according to the WHO Growth Standards for BMIz, as follows: Underweight: BMIz < −2, Normal weight: BMI in [−2; +1], Overweight: BMIz in ]+1; +2], Obesity: BMIz > +2 [37].

**Table 2:** Mean percentile per AEBQ subscale for each latent class.

AEBQ Subscales	Latent Class 1 Picky Eating	Latent Class 2 Disinterest towards Food	Latent Class 3 Food Neophilia	Latent Class 4 Emotional Eating	Latent Class 5 Food Attractiveness
Food Responsiveness/Enjoyment of Food	44.2	32.4	47.0	60.5	85.3
Emotional Overeating	53.3	16.6	52.1	78.3	71.8
Emotional Undereating	54.6	25.6	52.5	72.7	58.6
Food Fussiness *	71.8	45.7	25.2	52.4	48.5
Slowness in Eating	54.2	45.8	52.7	51.8	44.3

* After reversing items 12, 19, and 24.

**Table 3:** Associations between adolescent eating behavior patterns and maternal and adolescent sociodemographic and anthropometric characteristics using univariate multinomial logistic regression (reference category: “Disinterest towards food” pattern (Class 2)).

	Class 1 *Picky Eating*	Class 3 *Food Neophilia*	Class 4 *Emotional Eating*	Class 5 *Food Attractiveness*	*p*
	OR [95%CI]				
*Maternal characteristics*					
**Age at baseline (years)**	0.997 [0.979; 1.015]	0.995 [0.975; 1.015]	0.987 [0.966; 1.008]	**0.947 [0.927; 0.968]**	< 0.001
**Education (years of schooling)**	0.984 [0.963; 1.005]	1.003 [0.980; 1.026]	1.013 [0.988; 1.039]	**0.927 [0.904; 0.951]**	< 0.001
**Household income** **(euros per month)**	0.964 [0.900; 1.031]	1.015 [0.944; 1.091]	1.025 [0.948; 1.108]	**0.848 [0.778; 0.925]**	< 0.001
**BMI before pregnancy (kg/m^2^)**	1.017 [0.994; 1.039]	1.001 [0.976; 1.025]	0.996 [0.970; 1.024]	0.995 [0.968; 1.022]	0.405
*Birth characteristics*					
**Weight for gestational age**	0.906 [0.827; 0.992]	0.920 [0.833; 1.016]	0.876 [0.789; 0.972]	0.953 [0.854; 1.063]	0.098
*Adolescent characteristics*					
**Sex** (ref.: male)	**1.279 [1.065; 1.536]**	**1.614 [1.323; 1.970]**	**1.669 [1.344; 2.072]**	**2.137 [1.715; 2.661]**	< 0.001
**BMIz**	0.993 [0.919; 1.073]	**1.161 [1.066; 1.265]**	0.947 [0.864; 1.037]	1.055 [0.961; 1.157]	< 0.001
**Weight for gestational age**	0.906 [0.827; 0.992]	0.920 [0.833; 1.016]	0.876 [0.789; 0.972]	0.953 [0.854; 1.063]	0.098
**Birth order** (ref.: Youngest sibling)					0.063
Only child	1.014 [0.816; 1.259]	0.979 [0.769; 1.245]	1.003 [0.770; 1.307]	1.025 [0.789; 1.333]	
Oldest sibling	0.861 [0.682; 1.087]	1.092 [0.851; 1.401]	1.177 [0.898; 1.542]	0.969 [0.733; 1.280]	
Middle sibling	1.089 [0.685; 1.731]	1.409 [0.871; 2.280]	1.681 [1.017; 2.778]	2.054 [1.273; 3.317]	
**Beck Depression Inventory II (BDI-II)**	**1.028 [1.012; 1.045]**	**1.026 [1.008; 1.043]**	**1.054 [1.037; 1.072]**	**1.105 [1.088; 1.123]**	< 0.001

OR: odds ratio, CI: Confidence intervals, BMI: Body mass index, BMIz: Body mass index z-score. Bolded values are statistically significant (*p* < 0.05).

**Table 4:** Associations between adolescent eating behavior patterns and maternal sociodemographic and anthropometric characteristics using multivariable multinomial logistic regression models (reference category: “Disinterest towards food” pattern (Class 2)).

	Adjusted Model 1				
	Class 1 *Picky Eating*	Class 3 *Food Neophilia*	Class 4 *Emotional Eating*	Class 5 *Food Attractiveness*	*p*
	OR [95%CI]				
*Maternal characteristics*					
**Age at baseline (years)**	1.001 [0.979; 1.023]	0.997 [0.974; 1.020]	0.986 [0.961; 1.012]	0.973 [0.948; 0.998]	0.159
**Education (years)**	0.992 [0.964; 1.021]	1.003 [0.972; 1.035]	1.014 [0.980; 1.049]	**0.931 [0.899; 0.965]**	< 0.001
**Household income (€/month)**	0.981 [0.897; 1.073]	1.013 [0.921; 1.114]	1.030 [0.930; 1.141]	0.980 [0.878; 1.094]	0.883
**BMI before pregnancy (kg/m^2^)**	1.013 [0.989; 1.038]	1.007 [0.980; 1.034]	0.997 [0.968; 1.027]	0.981 [0.952; 1.011]	0.291

OR: odds ratio, CI: Confidence intervals, BMI: Body mass index, BMIz: Body mass index z-score. Adjusted for maternal characteristics (age at baseline, BMI before pregnancy, household income, and education). Bolded values are statistically significant (*p* < 0.05).

**Table 5:** Associations between adolescent eating behavior patterns and maternal and adolescent sociodemographic and anthropometric characteristics using multivariable multinomial logistic regression models (reference category: “Disinterest towards food” pattern (Class 2)).

	Adjusted Model 2				
	Class 1 *Picky Eating*	Class 3 *Food Neophilia*	Class 4 *Emotional Eating*	Class 5 *Food Attractiveness*	*p*
	OR [95%CI]				
*Maternal characteristics*					
**Age at baseline (years)**	0.998 [0.974; 1.022]	1.001 [0.975; 1.027]	0.988 [0.960; 1.017]	0.973 [0.945; 1.001]	0.325
**Education (years)**	0.995 [0.966; 1.025]	1.003 [0.971; 1.036]	1.017 [0.981; 1.053]	**0.943 [0.908; 0.979]**	0.005
**Household income (€/month)**	0.977 [0.893; 1.070]	1.005 [0.912; 1.107]	1.012 [0.912; 1.123]	0.964 [0.860; 1.081]	0.913
**BMI before pregnancy (kg/m^2^)**	1.016 [0.990; 1.043]	0.991 [0.963; 1.019]	1.007 [0.975; 1.039]	0.980 [0.948; 1.013]	0.184
*Birth characteristics*					
**Weight for gestational age**	0.919 [0.831; 1.016]	0.940 [0.842; 1.050]	0.899 [0.801; 1.009]	0.966 [0.851; 1.097]	0.354
*Adolescent characteristics*					
**Sex** (ref.: male)	**1.252 [1.019; 1.538]**	**1.527 [1.223; 1.906]**	**1.611 [1.265; 2.052]**	**1.672 [1.292; 2.163]**	< 0.001
**BMIz**	0.975 [0.889; 1.070]	**1.184 [1.069; 1.311]**	0.906 [0.811; 1.011]	0.978 [0.871; 1.097]	< 0.001
**Birth order** (ref.: Youngest sibling)					0.072
Only child	0.989 [0.765; 1.277]	0.888 [0.669; 1.178]	0.889 [0.651; 1.214]	0.859 [0.622; 1.188]	
Oldest sibling	0.914 [0.682; 1.225]	1.111 [0.813; 1.518]	1.122 [0.799; 1.574]	0.915 [0.635; 1.320]	
Middle sibling	1.136 [0.674; 1.915]	1.509 [0.880; 2.587]	1.934 [1.118; 3.346]	2.074 [1.186; 3.628]	
**Beck Depression Inventory II (BDI-II)**	**1.022 [1.004; 1.041]**	1.019 [1.000; 1.038]	**1.052 [1.033; 1.072]**	**1.098 [1.079; 1.118]**	< 0.001

OR: odds ratio, CI: Confidence intervals, BMI: Body mass index, BMIz: Body mass index z-score. Adjusted for Model 1 (maternal characteristics) plus adolescent characteristics (adolescent sex, BMIz, birth order, and depressive symptoms level). Bolded values are statistically significant (*p* < 0.05).

## Data Availability

Data are available upon request from the corresponding author, R.P.
